# Well-Trained Elders Have Antioxidant Responses and an Equal Magnitude of EIMD as Young Adults

**DOI:** 10.3390/ijerph19158889

**Published:** 2022-07-22

**Authors:** Eva Tékus, Nikolett Lilla Szénási, Edina Szabó, Zoltan Heckel, Tibor Mintál, Tamas Kőszegi, Tamas Atlasz, Zoltan Gazdag, Mark Váczi, Marta Wilhelm

**Affiliations:** 1 Institute of Sport Sciences and Physical Education, University of Pecs, H-7624 Pecs, Hungary; szenasi.nikolett@med.semmelweis-univ.hu (N.L.S.); szaboedina90@gmail.com (E.S.); heckel.zoltan@btk.ppke.hu (Z.H.); attam@gamma.ttk.pte.hu (T.A.); vaczi@gamma.ttk.pte.hu (M.V.); mwilhelm@gamma.ttk.pte.hu (M.W.); 2 Sports Medicine Center, Medical School, University of Pecs, H-7632 Pecs, Hungary; mintal.tibor@pte.hu; 3 Department of Anatomy, Medical School, University of Pecs, H-7624 Pecs, Hungary; 4 Doctoral School of Health Sciences, University of Pecs, H-7621 Pecs, Hungary; 5 Department of Laboratory Medicine, Medical School, University of Pecs, H-7624 Pecs, Hungary; koszegi.tamas@pte.hu; 6 János Szentágothai Research Center, University of Pecs, H-7624 Pecs, Hungary; 7 Department of General and Environmental Microbiology, Faculty of Sciences, University of Pecs, H-7624 Pecs, Hungary; gazdag@gamma.ttk.pte.hu

**Keywords:** antioxidants, eccentric, older adult, muscle damage, thiol

## Abstract

Aim The aim of the study was to investigate acute and chronic effects of a two-week eccentric concentric, dynamometric training concerning the time-course changes of blood antioxidant parameters (total antioxidant capacity, catalase enzyme activity, thiol concentration), and to compare the adaptability of young and older muscle to this type of training. Methods Seventeen moderately trained young and older men participated in this research. Subjects performed six eccentric concentric exercise bouts during the training period and maximal voluntary isometric contraction torque, plasma CK and intensity of muscle soreness were determined before and 24 h after the first exercise. During five testing sessions (baseline, 24 h, 48 h, week 1, week 2) the level of blood antioxidants were measured. Results No significant changes were registered in total antioxidant capacity and catalase enzyme activity for any time points; furthermore, no difference was found between groups during the training period. However, total thiol concentrations measured two weeks after the first exercise bout significantly differed between the young and elderly groups. Plasma CK and the subjective intensity of soreness elevated significantly 24 h following the first training, while maximal voluntary isometric contraction torque decreased at the same time. Conclusions Our results do not support previous findings that chronic, short-term eccentric concentric training programs enhance the antioxidant defense of well-trained older and young men. This type and setting of exercise did not cause a different time course of changes in the markers of exercise-induced muscle damage (EIMD) in the studied population. Subjects may already have adapted to maintain constant levels of antioxidants and isometric torque due to their active lifestyle.

## 1. Introduction

The relationship between regular physical activity and healthy ageing is well-known [1]. With ageing, the limitation of mobility, progressive reduction in force and skeletal muscle mass are measured, causing sarcopenia, which is associated with an increased chance of fractures, physical and cognitive disabilities and mortality [2,3].

Eccentric strength training with a higher mechanical force is more suitable to gain strength and enhance muscle hypertrophy [4] among both the young and elderly. Well-known consequences of single bout of eccentric exercise such as the magnitude of the plasma EIMD markers (such as creatine kinase enzyme activity—CK) is greater in younger adult, than in older adults [5]. In elderly people, short-term (two weeks) training may cause changes in muscle properties (strength and size increase) [6] and function (impaired muscle recovery); less effective metabolic adaptation [7] may differ from younger adults, which may be related to the prevention of fragility and sarcopenia [2]. However, the benefits of eccentric training [4] are not negligible even in old age. On the other hand, in elderly subjects, oxidative stress might play an essential role in a higher susceptibility to exercise-induced muscle damage, or longer recovery time and enhanced adaptation after an eccentric exercise bout [7].

Regular training was shown to influence the level of oxidants and adequate levels of total antioxidants positively to provide healthy aging and resulted in a higher level of total antioxidant capacity (TAC) [8]. The TAC of plasma may refer to the redox status of the human body [9]. Both acute exercise and regular training elevate TAC mainly by changing the regulation of transcription and the synthesis of enzymatic and non-enzymatic antioxidants in different cells [8]. Some data suggest that TAC remains unchanged during and after training sessions (in three sessions per week for 8 weeks aerobic training) [10], which might be explained by training intensity, frequency, timing, and other endogenous factors [8].

The antioxidant defense system of the human body is divided into two parts: enzymatic (superoxide dismutase, catalase, thiolcontaining enzymes, etc.) and non-enzymatic antioxidants (glutathione, uric acid, etc.) [11]. Catalase, belonging to the enzymatic antioxidants might spread out of the damaged muscle into the bloodstream after eccentric training. Two studies of young (18–23 years), healthy groups [12,13] focused on the effects of single bout eccentric, dynamometric exercises (5 sets of 15 eccentric maximal voluntary contractions) on the redox status of plasma, and found significantly higher serum catalase enzyme activity (CAT) at 24 and 48 h post-exercise in both genders.

Total thiol concentration (TTC) responses in blood plasma is poorly investigated during acute or chronic eccentric training, especially among elderly people, and the focus of recent studies has been on supplementation with a cysteine donor (like N-acetylcysteine) [14] to examine the performance-enhancing effects of thiol. Increase of plasma thiol [15] and protein thiols [16] concentrations were observed after a single bout of acute exercise among young adults. Rossi et al. [17] demonstrated similar resting TTC in different age-groups while an age-dependent reduction in total protein thiol levels as well as an elevation in cysteinylated and homocysteinylated plasma proteins was found, indicating that oxidative stress is increased with age.

A limited number of studies have been performed so far enrolling active, healthy elderly men and investigating the effects of short-term eccentric-concentric training on plasma redox status and the parameters of the plasma antioxidant system compared to young subjects.

In several studies, the beneficial effects of the long-term training [18] or single bout of exercise [19] on levels of antioxidants have been described among elderly. There is no evidence that short-term, eccentric concentric training has measurable effects on antioxidants in old age, while due to the functional and molecular changes in muscles [6] modifications in the antioxidant working mechanism are also expected. Considering the duration of the physical therapy (regardless of type—geriatric, orthopedic, cardiopulmonary, neurological-), this would be important information for physiotherapists, sport professionals, to know the short-term effect of training on redox balance among older adults. 

Therefore, the aim of the present study was to (i) investigate the acute and short-term chronic effects of a two-week eccentric concentric, dynamometric training on the time-course changes of antioxidant parameters (plasma total antioxidant capacity, plasma catalase enzyme activity, plasma thiol concentration), as well as to (ii) compare the adaptability of young and older muscle to this type of short-term training.

## 2. Materials and Methods

### 2.1. Participants

Seventeen healthy young and older adult men participated in the study. The young individuals (*n* = 8; age: 24.56 ± 2.40 years) were moderately trained physical education students, who did not participate in any competitive sports, but who regularly engaged in physical activities. They performed physical activity 8–10 h per week. Older adults (*n* = 9; age: 63.67 ± 5.34 years) were physically active in everyday routines and have not been involved in regular strength training for years. However, all subjects performed recreational sports activities (e.g., yoga, cycling, walking, swimming) regularly, at least 3 times a week. All subjects were requested to avoid any strenuous and unusual exercise one week prior to and during the study.

Exclusion criteria were smoking, dietary supplementation, and taking medications in the last three months before the study, and a current knee injury, previous hip surgery, and existing muscle pain. They were not suffering from any apparent acute or chronic illnesses. Subjects were asked to continue their usual diet and eating habits without modifying the frequency and quantity of their meals.

One week before the first measurement, subjects were acquainted with the testing equipment and protocol. Participants were given verbal and written information about the experiment, their anonymity was preserved and they signed an informed consent to participate in the study, which was approved by the Ethics Committee of our university (Permission number: 4817) in accordance with provisions of the Helsinki Declaration. 

### 2.2. Design and Procedures

**Figure 1:**
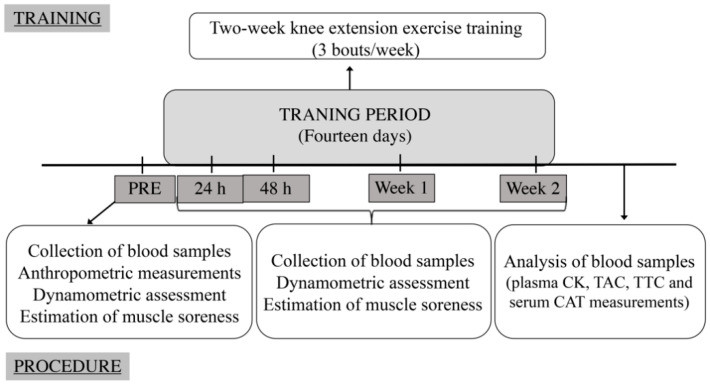
Training and study protocol during the experimental period CK = creatine kinase enzyme activity, TAC = total antioxidant capacity, TTC = total thiol concentration, CAT = catalase enzyme activity.

All subjects performed six eccentric concentric knee extension exercise bouts during a two-week training period. Five test sessions (baseline, 24 h, 48 h, 1 week as well as 2 weeks after bout 1) were implemented. The following variables were measured: anthropometric parameters (height, body weight, body mass index) only at baseline, knee extension torque at maximal voluntary isometric contraction (MVC), level of blood antioxidants (TAC, TTC, CAT) plasma CK and the intensity of subjective muscle soreness. All exercises and measurements were performed between 9.00 and 12.00 a.m.; blood collection was always performed first (Figure 1).

### 2.3. Anthropometric Measurements

Baseline anthropometric parameters were measured. The body mass index (BMI) of the participants was calculated using body height (Martin type anthropometer) and body mass (Beurer BG-55 scale, Beurer GmbH, Ulm, Germany) data of subjects.

### 2.4. Quadriceps MVC Torque and Intensity of Muscle Soreness

Quadriceps MVC (maximal voluntary isometric contractions) torque was measured in a seated position on a Multicont II isokinetic device (Mediagnost, Budapest and Mechatronic Ltd., Szeged, Hungary) at onset and 24 h after the first exercise bout. Subjects performed three MVCs with the right quadriceps at 70° knee flexion, within the range of the optimal knee angles, with two minutes recovery between trials. They were instructed to generate the highest possible torque, and the value of the best trial was used for statistical tests.

The subjective soreness of the knee extensor muscle group was estimated using a visual analog soreness scale (consisting of a 50 mm line, where 0 is no pain at all, and 50 is extreme pain) marked after the MVC test contractions at each testing session.

### 2.5. Blood Sampling and Analyses

Subjects were instructed not to eat 12 h before blood collection. Venous blood samples were taken using EDTA-containing and plain, Vacutainer tubes at baseline, and at 24 h, 48 h, 1 week and 2 weeks following the first exercise bout. Blood samples were always taken before exercise or test sessions. After clotting, samples were centrifuged at 1500× *g* for 10 min; plasma aliquots in 1.5 mL Eppendorf tubes were stored at −70 °C until analyses.

For CK enzymatic activity measurement, a kinetic optimized UV test was used in the accredited routine clinical laboratory of the Department of Laboratory Medicine by an automated clinical chemistry analyzer (Cobas Integra 400 Plus, Roche Diagnostics, Hungary) with an interassay precision of less than 7% for the coefficient of variation. The reference range of CK was 0–200 U/L.

The determination of plasma TAC was performed according to Lewinska et al. [20] and TTC was measured by Lewinska and Bartosz [21]. The specific activity of catalase was assessed by means of a well-established colorimetric assay [12]. The protein content of the plasma was measured by a modified Lowry method.

### 2.6. Dynamometric Exercise Training

Subjects performed a two-week-long eccentric concentric dynamometric exercise training with three scheduled exercise bouts per week. These bouts consisted of maximal effort knee extensions performed on the same dynamometer, which was used for the MVC tests.

After 5 min warming up on cycling ergometer (Ergoline 900, Ergometrics, Germany) and stretching the knee extensors and hip flexors, subjects performed 4 sets of 15 repetitions maximal eccentric concentric contractions with the right limb. Contractions were executed between 20 and 80° angles of the knee, at a 60°·s^−1^ constant angular velocity. A 1 s rest was provided between contractions and 2 min between sets.

### 2.7. Statistical Analyses

For evaluating the normality of data, the Kolmogorov-Smirnov test was used. Each variable showed a normal distribution, therefore a two-way (group by time) repeated measures analysis of variance was applied. One-way repeated measures ANOVA tests were used with Bonferroni post-hoc analyses to investigate the differences among the two or the five measurement times. Significant differences between the two groups were determined with independent samples *t*-test. For primary outcome variables, pre-post change and pre-post percentage change were calculated between time points (pre-24 h, pre-48 h, pre-week 1, pre-week 2). The statistical power was determined using the G power 3.1 software. Values are reported as mean ± standard error of the mean (SEM). The level of significance was set at *p* < 0.05.

## 3. Results

Table 1 presents anthropometric data and the decimal age of the experimental groups. In accordance with the normal ageing processes, weight and BMI were significantly lower among younger subjects, while the mean height was nearly equal in both groups. The statistical power of the repeated measure ANOVA was found to be between 0.9 and 1.0 for the main outcome variables (MVC torque, plasma CK, subjective intensity of muscle soreness, plasma TTC).

There was no significant group by time interactions concerning plasma CK, MVC torque, and intensity of muscle soreness (Table 2), suggesting that changes were similar in both groups. Significant (*p* < 0.05) time main effects were found in 24 h after the exercise bout in measured CK, and significant MVC torque changes were also measured at 24 h after the training when compared to baseline values, suggesting that the two groups responded similarly. The subjective intensity of soreness applying a visual analog soreness scale was significantly (*p* < 0.05) higher at 24 h after the first exercise bout when compared to baseline values.

**Figure 2:**
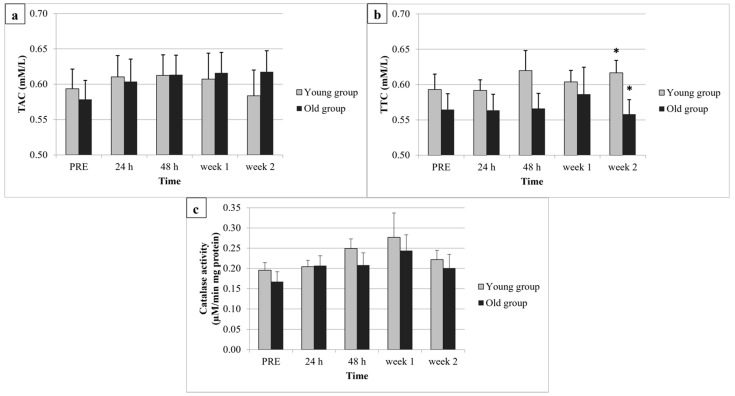
Plasma total antioxidant capacity (**a**), thiol concentration (**b**) and serum catalase (**c**) during the two-week training period among old and young groups. MEAN and SEM were calculated from four independent experiments/samples. TAC = total antioxidant capacity, TTC = total thiol concentration, * Significant (*p* < 0.05) difference between the two groups.

Neither group by time interaction nor significant group and time main effects were found in plasma TAC (Figure 2a) and serum CAT (Figure 2c), suggesting that these two antioxidant parameters were not sensitive to the two-week eccentric concentric training, and were independent of age.

A significant group by time interaction was observed in plasma TTC. The post-hoc analysis revealed a significant difference (*p* = 0.049) at two weeks following the first exercise bout between the two groups: the young group had a significantly higher TTC value. Meanwhile, participants (old and young groups also) had similar changes (absolute, percentage) in thiol concentrations between each measurement time. In other time points, we did not find differences in plasma thiol levels (Figure 2b) suggesting that the two groups responded differently to the applied training.

## 4. Discussion

Our research focused on the age-specific effects of a two-week eccentric concentric dynamometric training on the time-course changes in conventional muscle damage markers and antioxidant parameters.

In the present study, six exercise bouts induced no change in plasma TAC and serum CAT in either of the experimental groups, suggesting age groups have similar antioxidant function. However, we demonstrated that older adults respond with lower plasma TTC measured two weeks after the first exercise bout, compared with the younger ones. As it was expected, plasma CK and the subjective intensity of soreness elevated at 24 h following the first training bout in both groups, while MVC torque decreased at the same time without any age-specific changes.

In this study, similar responses in muscle damage markers (force reduction, plasma CK, delayed onset muscle soreness) were detected both in young and old people at onset and 24 h after the first exercise. We supposed that well-trained individuals of different ages with almost the same fitness level (concerning the baseline MVC torque) suffered a similar magnitude of muscle damage as a result of an eccentric concentric exercise bout. In agreement with literature data, in the present study, an increase of CK, delayed onset of muscle soreness symptoms, and strength loss following the first exercise bouts were found due to EIMD in both groups [22]. Others described elevated EIMD markers (higher force reduction, plasma CK, lactate dehydrogenase enzyme activity, muscle soreness) among older women and men after an eccentric exercise bout [22,23]. Otherwise, in old age, redox imbalance may cause a higher susceptibility to EIMD, or a less-effective recovery and enhanced adaptation after an eccentric exercise bout [7]. It is well-known that, the level of antioxidants is positively influenced by long-term training [18] or a single bout of exercise [19] among elderly. There are no data as to whether the short-term, eccentric concentric training has measurable effects on antioxidants in old age; due to the functional and molecular changes in muscles [6], modifications in antioxidant working mechanism are also expected.

There was no difference in antioxidant (plasma TAC, serum CAT) responses between groups in any time points, suggesting this result was independent of age. Supposedly, both older and young might have already adapted, resting antioxidant levels due to their active lifestyle, which might lead to similar level of antioxidants in the groups. However, Chen et al. [24] described the age-dependent adaptation of the antioxidant system to oxidative stress, and found aged muscles more susceptible to oxidative damage. Although, they did not study trained muscles’ relation to changes in antioxidants function during ageing.

In our study, plasma TAC and serum CAT did not change after the first exercise bout, as well after the two-week eccentric training, suggesting that it was not sensitive to the applied training. Our findings are opposite to some literature data concerning the effects of different training programs of oxidative stress markers among the ageing population. Some have reported a decrease [18,25], while others have found no changes in the values [26]. The reasons for these contradictory findings might be the numerous influencing factors of oxidative stress, such as muscle mass and the types of groups recruited, the modes of contraction, exercise intensity and duration, and the exercising population [27]. In our experimental setting (short-term training), there was no change in antioxidant levels from baseline values, regardless of age, suggesting that the adaptation of antioxidant mechanisms would require a longer time.

In general, the effectivity of regular training was proven with the increase of total antioxidant concentrations and the reduction of oxidants [8], and the decreased susceptibility to EIMD and dysfunction [23]. It is an important question as to whether the TAC of well-trained older persons would rise after an acute exercise bout. Gwozdzinski et al. [15] found an increase of plasma antioxidant capacity and plasma total thiols one hour after a single bout of maximal exercise on a cycling ergometer among untrained, young males. The plasma TAC level was influenced by physiological, psychological, pathological, environmental and nutritional factors [9]. Theodorou et al. [13] observed an elevation in the plasma TAC and CAT of young, healthy men after an isokinetic eccentric contraction session. Resting plasma TAC and CAT were unchanged in recreationally trained and untrained groups after 4 weeks of eccentric training performed on an isokinetic dynamometer [28].

In our study, the age-related difference in baseline TTC and changes between time points in thiol concentration were not detected, but the applied eccentric concentric training program may cause different TTC responses among young and older humans. Age-related decreases (mainly from 60–70 years) in total protein thiols level are accompanied by the elevation of S-thiolated protein concentrations, suggesting that the target proteins of oxidative stress are often thiols [17]. Older individuals had a lower TTC, referring to the lower antioxidant defense. Contrary to the previous studies, we assumed that by measuring individuals with almost the same fitness level (concerning similar MVC torque) in different age groups, similar resting antioxidant levels would be detected. Others described greater oxidative damage accumulated in the aging body during short-term training, with the balance between oxidative and antioxidant processes having shifted toward oxidants, as oxidative stress and damage increase with age [29]. However, reduced oxidative stress (measured by activity of antioxidant enzymes) may be related to physical fitness among healthy elderly individuals [30].

A limitation of this work was that the investigated subjects were a small group of elderly men with an outstanding fitness level; therefore, the conclusions of the study can only be applied to similar, highly trained older adults.

## 5. Conclusions

In conclusion, we show similar antioxidant responses (except plasma TTC) in young and older adults after a chronic, short-term eccentric concentric training program, either because of the similar fitness level (concerning the baseline isometric torque) or EIMD response. Furthermore, we supposed that antioxidant responses cannot be evoked in as short as two weeks. This type and setting of exercise did not cause a different time course of changes in EIMD markers (plasma CK, MVC torque, intensity of muscle soreness) in the studied population. Subjects may already have adapted baseline levels of antioxidants and baseline isometric torque due to their active lifestyle. These findings support the importance of regular training, especially in old ages.

## Figures and Tables

**Table 1:** Anthropometric parameters of the two experimental groups.

	Young Group	Old Group
Decimal age (years)	24.75 ± 0.88	63.67 ± 1.78
Height (m)	1.76 ± 0.03	1.76 ± 0.02
Weight (kg)	71.00 ± 2.28 *	81.22 ± 3.05 *
BMI (kg/m^2^)	23.00 ± 0.62 *	26.21 ± 0.75 *

* Significant (*p* < 0.05) difference between the two groups. MEAN ± SEM.

**Table 2:** Indirect muscle damage markers before and 24 h after the first exercise bout (MEAN ± SEM).

	PRE			24 h		
	Young Group	Old Group	Total	Young Group	Old Group	Total
MVC torque (Nm)	249.56 ± 14.23	209.72 ± 14.53	228.47 ± 11.06 *	219.13 ± 16.98	179.23 ± 13.38	198.00 ± 11.47 *
Plasma CK (U/L)	163.14 ± 57.12	103.44 ± 13.92	129.56 ± 26.22 *	549.14 ± 170.38	374.44 ± 96.05	456.76 ± 94.32 *
Intensity of muscle soreness	0.00 ± 0.00	0.00 ± 0.00	0.00 ± 0.00 ^#^	24.00 ± 7.16.00	13.11 ± 3.10	18.24 ± 3.86 ^#^

* Significant (*p* < 0.01) difference between combined values of the two groups. MEAN ± SEM. ^#^ Significant (*p* < 0.05) difference between combined values of the two groups. MEAN ± SEM.

## Data Availability

The data that support the findings of this study are openly available in FigShare at https://figshare.com/articles/dataset/Well-trained_elders_have_antioxidant_responses_and_equal_magnitude_of_EIMD_as_young_adults_-_dataset/14959398/1 (accessed on 12 July 2021).
